# Toward Enhanced Antioxidant and Protective Potential: Conjugation of Corn Cob Xylan with Gallic Acid as a Novel Approach

**DOI:** 10.3390/ijms25052855

**Published:** 2024-03-01

**Authors:** Isabelle Luna Oliveira Dantas-Berto, Rony Lucas Silva Viana, Mayara Jane Campos de Medeiros, Leonardo Thiago Duarte Barreto Nobre, Ana Carolina Luchiari, Valquíria Pereira Medeiros, Weslley Souza Paiva, Raniere Fagundes Melo-Silveira, Hugo Alexandre Oliveira Rocha

**Affiliations:** 1 Graduate Program of Health Sciences, Federal University of Rio Grande do Norte (UFRN), Natal 59078-970, RN, Brazil; isabelleluna@outlook.com.br; 2 Laboratory of Biotechnology of Natural Polymers (BIOPOL), Graduate Program of Biochemistry and Molecular Biology, Bioscience Center, Federal University of Rio Grande do Norte—UFRN, Natal 59078-970, RN, Brazil; rony_lucas@hotmail.com (R.L.S.V.); leo.dnobre@gmail.com (L.T.D.B.N.); wdspaiva@gmail.com (W.S.P.); ranierefagundes@hotmail.com (R.F.M.-S.); 3 Coordination Chemistry and Polymers Laboratory (LQCPol), Department of Chemistry, Institute of Chemistry, Federal University of Rio Grande do Norte (UFRN), Natal 59078-970, RN, Brazil; mayarajane20049@hotmail.com; 4 Laboratory of Ornamental Fish, Federal University of Rio Grande do Norte, Natal 59078-970, RN, Brazil; luchiarilab@gmail.com; 5 Department of Biochemistry, Federal University of Juiz de Fora, Juiz de Fora 36036-900, MG, Brazil; valmedeiros@gmail.com

**Keywords:** modified polysaccharides, oxidative stress, oxidative damage, zebrafish

## Abstract

Maize ranks as the second most widely produced crop globally, yielding approximately 1.2 billion tons, with corn cob being its primary byproduct, constituting 18 kg per 100 kg of corn. Agricultural corn production generates bioactive polysaccharide-rich byproducts, including xylan (Xyl). In this study, we used the redox method to modify corn cob xylan with gallic acid, aiming to enhance its antioxidant and protective capacity against oxidative stress. The conjugation process resulted in a new molecule termed conjugated xylan–gallic acid (Xyl-GA), exhibiting notable improvements in various antioxidant parameters, including total antioxidant capacity (1.4-fold increase), reducing power (1.2-fold increase), hydroxyl radical scavenging (1.6-fold increase), and cupric chelation (27.5-fold increase) when compared with unmodified Xyl. At a concentration of 1 mg/mL, Xyl-GA demonstrated no cytotoxicity, significantly increased fibroblast cell viability (approximately 80%), and effectively mitigated intracellular ROS levels (reduced by 100%) following oxidative damage induced by H_2_O_2_. Furthermore, Xyl-GA exhibited non-toxicity toward zebrafish embryos, offered protection against H_2_O_2_-induced stress, and reduced the rate of cells undergoing apoptosis resulting from H_2_O_2_ exposure. In conclusion, our findings suggest that Xyl-GA possesses potential therapeutic value in addressing oxidative stress-related disturbances. Further investigations are warranted to elucidate the molecular structure of this novel compound and establish correlations with its pharmacological activities.

## 1. Introduction

Antioxidants are compounds capable of neutralizing harmful molecules like reactive oxygen species (ROS), which can harm cells and contribute to the onset of chronic conditions such as cancer, Alzheimer’s disease, arteriosclerosis, diabetes, and obesity. Moreover, antioxidants play a vital role in preserving the stability and integrity of various biological molecules while shielding cells from harm caused by exposure to toxins, radiation, or pollutants [1]. In general, antioxidants have demonstrated promising health advantages, including a reduced risk of specific diseases and the deceleration of the aging process [2].

The ideal antioxidant should possess the ability to efficiently neutralize a broad spectrum of free radicals and other reactive species. It should demonstrate resilience against chemical degradation, thereby retaining its antioxidant efficacy over time. Furthermore, it should be deemed safe for consumption by humans and animals, exhibiting high bioavailability, functioning effectively in diverse environments, including both aqueous and lipid-based contexts, and maintaining its effectiveness across a range of pH levels. Additionally, it should be economically viable to produce, ensuring accessibility to a wide and diverse population [3].

It is important to note that no single antioxidant compound possesses all these characteristics. Consequently, researchers are constantly exploring new antioxidant compounds that offer potential health benefits and can be used in conjunction with other antioxidants. In this context, some polysaccharides, including agarose, glucans, and cellulose, undergo chemical modifications with the objective of obtaining polymers with enhanced antioxidant potential, thus broadening the application of these molecules [4,5]. One such example is the conjugation of chitosan with gallic acid. This conjugate exhibited improved water solubility and superior antioxidant activity compared with chitosan and gallic acid when used individually [6]. However, as far as we know, there are no papers that demonstrate the conjugation of gallic acid with corn cob xylan, nor is it known if there are benefits in this conjugation.

Maize is the second most widely produced crop globally, yielding approximately 1.2 billion tons [7]. Its primary by-product is corn cob, with every 100 kg of corn containing 18 kg of cob. Several studies have already identified potential uses for corn cobs, including solid biofuel production (pellets) [8], ethanol production [9], and the extraction of nanocrystalline cellulose [10]. Furthermore, in other studies, corn cobs subjected to physical or chemical processes resulting in slight structural modifications have shown promise as environmental bioremediation agents for absorbing pesticides such as atrazine and imidacloprid [11], as well as various metals, including uranium, lead, cadmium, and nickel [12,13]. However, a significant portion of the corn cob mass produced is currently being discarded, leading to environmental damage [14].

Hence, it is crucial to explore various applications for corn cobs and their biomolecules to mitigate or even prevent their underutilization. Among these biomolecules, xylan deserves special mention [15].

Xylan is a xylose-rich polysaccharide extracted from corn cobs. Xylan shows immunomodulatory properties [16], as well as antimicrobial, antioxidant, anticoagulant [17], and antiproliferative effects against tumor cells [18]. Moreover, the xylan extraction method is already well-established [16]. However, to the best of our knowledge, there is no record of xylan being used in commercial biomedical applications.

Gallic acid, a natural polyphenol found in various plants, including vegetables [19,20], possesses structural characteristics that render it a compelling candidate for conjugation. Its carboxyl region facilitates attachment to other molecules. Commercially, gallic acid can be obtained through the hydrolysis of tannin-rich materials, such as gallotannins, for instance, pentagalloylglucose, which naturally occur in plants. This hydrolysis process can be achieved using either chemical or enzymatic means. Alternatively, gallic acid production can be facilitated via microbial fermentation, an economically viable and environmentally safe process. In this method, the microbial enzyme tannase, derived predominantly from fungi or bacteria, catalyzes the cleavage of ester bonds, liberating gallic acid molecules. Consequently, these process renders it a cost-effective product [21]. Furthermore, it exhibits low toxicity [22], making it suitable for diverse industrial applications. Nevertheless, phenolic compounds, including gallic acid, sourced from food face limited bioavailability due to their poor absorption, rapid metabolism, and subsequent excretion [23,24], despite their inherent antioxidant capacity.

The conjugation of gallic acid with xylan can effectively enhance the antioxidant properties of xylan. Furthermore, the conjugation of xylan with gallic acid has the potential to increase the bioavailability of gallic acid. Therefore, the conjugation of these two molecules introduces a novel biomaterial that synergizes the functionalities of gallic acid and xylan, thus opening new avenues for xylan’s application. This advancement adds value to corn cobs by offering a solution to address the environmental damage resulting from their current underutilization.

## 2. Results and Discussion

### 2.1. Total Phenolic Compounds, Total Sugar, and Protein of Xyl and Xyl-GA

The extraction process utilized in this study resulted in a xylan conversion efficiency of 37.0% ± 2.0% relative to the mass of corn cob meal utilized. The extraction methods yielded xylan quantities comparable to those reported by Viana et al. [25], and surpassed the yields obtained by Ebringerová and colleagues (15%) [16]. Notably, the latter group did not utilize an alkaline solution during the extraction process, which may have contributed to their lower yield.

Table 1 displays the results obtained from the chemical analysis of xylan. Considering the quantities of sugar, proteins, and phenolic compounds, the extraction yielded a sample rich in sugar (97.4%) and low in contaminants, with phenolic compounds at 0.8%. Table 1 also presents the chemical composition of Xyl-GA. In comparison to Xyl, it is evident that the sugar content in Xyl-GA was slightly lower, although not significantly different. Conversely, the phenolic compound content in Xyl-GA was significantly higher than in Xyl.

The conjugation of xylan with gallic acid had not been previously demonstrated, making it impossible to directly compare the results in Table 1 with those of other studies. However, when the same gallic acid conjugation method was applied to chitosan [26], laminarin [27], fucoidan [28], and dextran [6], the authors confirmed the success of the conjugation by observing an increase in phenolic compounds within the polysaccharide composition using the Folin–Ciocalteu method, which was also used in this study. Therefore, it is plausible to deduce that the notable rise in phenolic compounds in Xyl-GA corresponds to gallic acid molecules bonded to xylan.

### 2.2. Analysis of the FTIR Spectrum of Xyl and Xyl-GA

**Figure 1:**
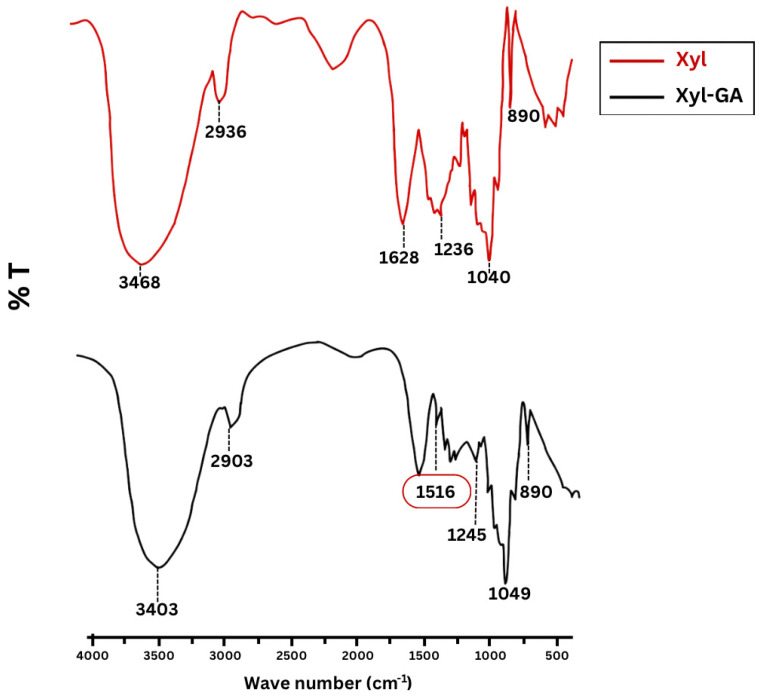
Infrared spectrum within the range of 4000–500 cm^−1^ for Xyl and Xyl-GA. The band around 1516 cm^−1^ (red circle) indicating the presence of an aromatic ring linked to the polysaccharide.

Figure 1 displays the FTIR spectra in the range between 2000 cm^−1^ and 400 cm^−1^, where the primary identification bands for Xyl and Xyl-GA are situated.

The bands characteristic of polysaccharides are evident in both spectra at specific wavenumbers: 3460–3400 cm^−1^ (O–H stretching and intramolecular hydrogen bonds); 2900–2940 cm^−1^ (C–H symmetric and asymmetric stretching); 1600 cm^−1^ (C=O stretching of monosaccharides); and bands in the 1200–1000 cm^−1^ region, which is dominated by ring vibrations overlapped with stretching vibrations of C–OH side groups and C–O–C glycosidic band vibration. A specific band appears at 1040–1050 cm^−1^ (stretching of the C-OH bond) [17]. This band dominates the xylan spectrum with (1–4)-backbone; moreover, the anomeric region of (1–4)-xylan is assigned at 890 cm^−1^ in accordance with the IR results in xylobioside models [29]. 

In the Xyl-GA spectrum, there is a notable increase in the band intensity around 1516 cm^−1^, indicating the presence of an aromatic ring linked to the polysaccharide [30]. Additionally, the band at 1628 cm^−1^, observed in the Xyl spectrum, shifts in the Xyl-GA spectrum. This shift occurs due to an overlap between the bands of the C=O group of monosaccharides and the C=O group of the ester and the C=C vibrations on the aromatic rings. This ester formation takes place when the carboxyl group of gallic acid bonds with the hydrolxyl group of the polysaccharide [30]. This shift also suggests the presence of gallic acid bound to the polysaccharide. Taken together, the existence of these two bands supports the data on phenolic compound quantification and signifies the conjugation of gallic acid with xylan.

### 2.3. In Vitro Evaluation Xylan’s Antioxidant Activity

The antioxidant activity of xylan has been demonstrated by several authors [17,18,25,31,32]. Additionally, it has been shown that modifications involving gallic acid can enhance the antioxidant activities of polysaccharides [6,25,27,28]. Furthermore, the efficacy of antioxidants is assessed based on the stages of the oxidative process at which they can exert their effects, and the quality of antioxidants is evaluated based on the number of stages they can act upon [33,34]. To compare the antioxidant activity of Xyl-GA with that of Xyl, different tests, both in vitro and in vivo, were conducted. These tests collectively assessed the effectiveness of the agents at various stages of the oxidative process.

#### 2.3.1. Copper Ion Chelating Ability

The accumulation of excessive copper levels in the body, whether resulting from overconsumption, toxicity, or genetic anomalies such as those seen in Wilson’s disease [35] and Menkes syndrome [36], can lead to oxidative stress. This oxidative stress ultimately triggers cell apoptosis in various organs, including the brain, kidneys, and corneas [37]. To address this issue, strategies such as reducing copper intake and utilizing chelating agents (e.g., D-penicillamine or trientine) to enhance urinary copper excretion are commonly recommended [38]. Nevertheless, there remains an ongoing demand for the development of novel chelating agents capable of potentially replacing current options. Within this context, the copper chelating activity of Xyl and Xyl-GA was evaluated.

**Figure 2:**
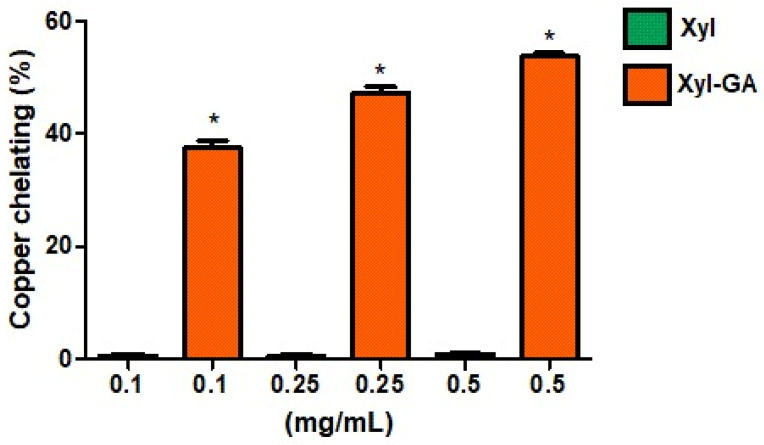
Analyses of the capacity to chelate copper ions. * Indicates a significant difference between Xyl and Xyl-GA samples at the same concentration (*p* < 0.001).

The copper chelation activity of Xyl and Xyl-GA was assessed as outlined in the Methods Section, and the results are depicted in Figure 2. It is evident that Xyl did not exhibit any chelation activity under the tested conditions. In contrast, Xyl-GA demonstrated chelation activity at all evaluated concentrations, achieving approximately 55% chelation at the highest concentration tested (0.5 mg/mL). It is noteworthy that the copper chelation activity of gallic acid at a concentration of 1.0 mg/mL was observed to be merely 10% ± 1.4. In essence, this indicates that even when compared at double the concentration of Xyl-GA, gallic acid exhibits significantly lower activity than this conjugated polysaccharide.

The incorporation of gallic acid into the structure of other polysaccharides, such as laminarin [27] and fucoidan [28], has also been shown to enhance their copper chelation activity. The effectiveness of copper chelation depends on a combination of structural features, including functional groups capable of forming coordinated bonds with copper ions, structural flexibility, and the presence of multiple donor atoms (e.g., oxygen, sulfur, and nitrogen). Furthermore, the molecular structure should be well-suited to the geometry of copper ions. These essential characteristics are found in gallic acid, which is recognized as an effective copper chelator [39].

However, it is important to note that the addition of gallic acid to the structure of a polysaccharide does not guarantee that the resulting compound will exhibit greater copper chelation activity than the original polysaccharide. For instance, when chitosan was conjugated with gallic acid, its copper chelation activity did not exhibit an increase [6].

Wang et al., 2013 [40] proposed that antioxidant activity is not solely attributed to the addition of functional groups but also depends on the structural conformation of the polysaccharide after modification. Therefore, the data obtained in this study suggest that it is not solely the presence of gallic acid that enhances the chelating activity of the formed conjugate but likely the way this substituent is distributed throughout the polysaccharide molecule and how it influences its conformation. This aspect appears to be a crucial factor for the polysaccharide conjugated with gallic acid to exhibit copper chelation activity.

Studies investigating the conformational changes in xylan following its conjugation with gallic acid are beyond the scope of this study. Nevertheless, the data suggested that Xyl-GA may be categorized as a preventive antioxidant, as it can chelate copper and, thereby, mitigate the formation of reactive species, including the hydroxyl radical.

#### 2.3.2. Evaluation of Hydroxyl Radical Scavenging Activity of Xylans

The hydroxyl radical is exceptionally reactive among all reactive oxygen species. It has the capability to abstract hydrogen atoms from thiol-containing biological molecules, leading to the formation of sulfur radicals. These sulfur radicals can subsequently react with oxygen, giving rise to the generation of oxysulfate radicals, which have the potential to inflict damage upon biological molecules [41]. That is why it is important to identify molecules with a strong hydroxyl radical scavenging activity.

**Figure 3:**
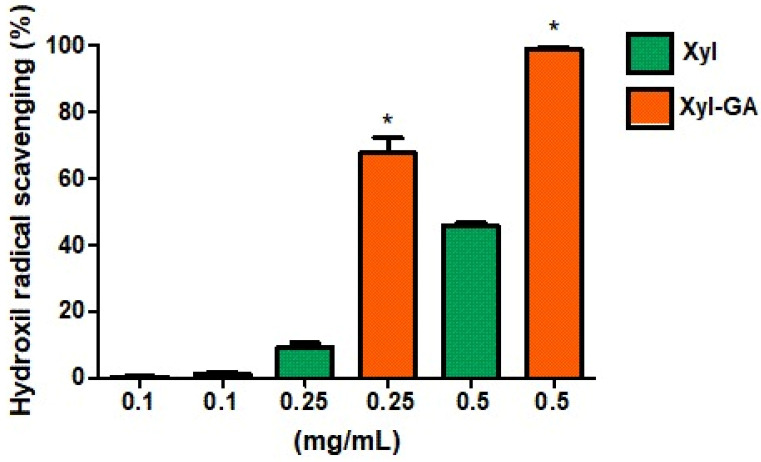
In vitro hydroxyl radical scavenging activity of Xyl and Xyl-GA. * (*p* < 0.001) indicates a significant difference between the same concentrations of Xyl and Xyl-GA samples.

To assess the antioxidant mechanisms of Xyl and Xyl-GA, a test was conducted to evaluate their ability to scavenge hydroxyl radicals using the method described in the Materials and Methods Section. The data are shown in Figure 3.

It is worth noting that both tested samples exhibited the capability to scavenge hydroxyl radicals, as depicted in Figure 3. However, Xyl-GA displayed significantly higher activity at the two highest concentrations assessed (0.25 and 0.5 mg/mL) compared with Xyl. Moreover, at a concentration of 0.5 mg/mL, Xyl-GA exhibited nearly 100% hydroxyl radical scavenging.

Based on the findings presented in Table 1, it was determined that Xyl-GA comprises approximately 2.4% phenolic compounds. Consequently, at a concentration of 0.5 mg/mL Xyl-GA, the presence of gallic acid was estimated to be around 0.012 mg/mL. Subsequently, the antioxidant activity of gallic acid at this concentration (0.012 mg/mL) was evaluated, revealing a hydroxyl radical scavenging value of 5.1% ± 0.2. Notably, upon increasing the concentration of gallic acid to 1.0 mg/mL, a disproportionate enhancement in the observed activity was noted, with a hydroxyl radical scavenging value of 10.0% ± 1.7. These data demonstrate that the observed activity with Xyl-GA (0.5 mg/mL) does not solely result from the sum of the activities of its individual components, namely, xylan and gallic acid. Instead, it predominantly arises from the conjugation of these two molecules.

Hromádková et al. [42] suggest that phenolic components, particularly phenolic acids, play an essential role in the overall radical scavenging capacity of xylans and xylooligosaccharides from wheat bran. This also appears to hold true for corn cob xylan, as the conjugation of this xylan with a small quantity of gallic acid was sufficient to enhance its activity.

The conjugation of gallic acid with other polysaccharides has also been shown to enhance its ability to scavenge hydroxyl radicals. For instance, a fucoidan, when evaluated at 0.5 mg/mL, saw its activity increase by 30% following the conjugation process with gallic acid [28]. However, the conjugation of gallic acid with a polysaccharide does not always guarantee the transfer of this activity to the resulting compound. For instance, laminarin derived from the seaweed *Lobophora variegata* was conjugated with gallic acid but did not exhibit hydroxyl radical-scavenging activity, even at high concentrations (2.0 mg/mL) [27].

Hou et al., 2012 [43] reported that the molecular mass and spatial structure of polysaccharides are determining factors for their hydroxyl radical scavenging ability. Consequently, it is presumed that the conformation adopted by Xyl-GA after conjugation enabled its hydroxyl radical scavenging activity. Furthermore, these findings suggest that Xyl-GA is an antioxidant compound that directly neutralizes reactive species such as hydroxyl radicals.

#### 2.3.3. Capacity to Donate Electrons (e^−^)

Reducing Power and Determination of Total Antioxidant Capacity (TAC) are assays that evaluate antioxidant activity by analyzing the capacity of a sample to donate e^−^ in pH close to neutral and acidic, respectively.

##### Reducing Power

**Figure 4:**
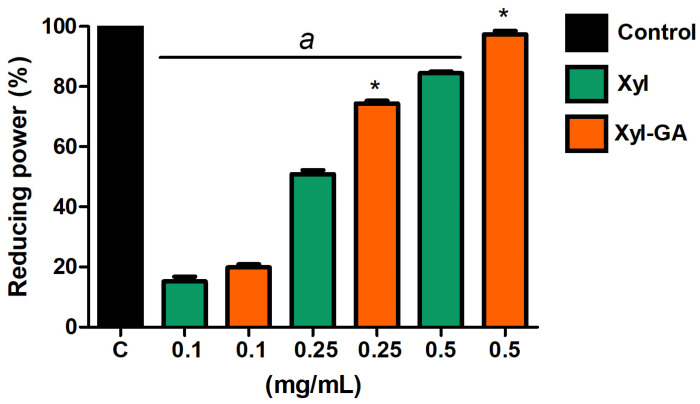
Reducing powers of Xyl and Xyl-GA. * Represents significant differences between Xyl and Xyl-GA at the same concentration (*p* < 0.001). “a” represents a significant difference between the samples and control (*p* < 0.001).

Both Xyl and Xyl-GA exhibited reducing power (Figure 4). However, at the highest concentrations tested (0.25 and 0.5 mg/mL), Xyl-GA showed significantly higher activity than Xyl. In addition, Xyl-GA showed a reducing power of almost 100% at a concentration of 0.5 mg/mL. On the other hand, GA (0.012 mg/mL and 1.0 mg/mL) showed no activity in this test.

##### Determination of Total Antioxidant Capacity

The data obtained from the determination of the Total Antioxidant Capacity (TAC) of the samples revealed that Xyl-GA exhibited a TAC value of 93.2 ± 2.3 mg of ascorbic acid equivalent (AA) per gram of sample, whereas Xyl had a value of 64.4 ± 1.8 mg AA/g of the sample, and GA had a value of 22.5 ± 4.3 mg AA/g of the sample. In essence, akin to the outcomes observed in other assays, the conjugation with gallic acid resulted in increased activity in Xyl-GA compared with Xyl or GA alone, with the activity being approximately 45% and 75% higher, respectively, in this instance.

The data show that Xyl-GA possesses the capability to donate electrons under neutral pH conditions (as observed in the reducing power test) and acidic pH conditions (as evident in the TAC assay). This characteristic has been previously observed in chitosan conjugated with gallic acid [26,32]. While fucoidan [36] and dextran [6] enhanced their electron donation ability in the reducing power test, they did not exhibit a similar improvement in the TAC test.

Gallic acid binds the polysaccharide through its carboxyl group, forming an ester with the polysaccharide at the end of the process [44] This leaves the hydroxyls of the aromatic ring free to facilitate the reduction of reactive species. It is believed that the para-substituted -OH groups form hydrogen bonds with the meta-substituted -OH group, resulting in a lower hydrogen bond dissociation enthalpy and, consequently, they can stabilize this group after it has donated one of its electrons [45]. However, the electron donation efficiency of gallic acid depends on its steric freedom, which in turn depends on its substituent [46]. 

This demonstrates that Xyl-GA can, under two different pH conditions, provide the necessary environment for gallic acid to have steric freedom. Consequently, this makes Xyl-GA an effective electron donor in the two tests mentioned above. These two tests were performed at different pH values, which can change the polysaccharide conformation and, consequently, the steric freedom of the GA.

### 2.4. Evaluation of Xylan’s Antioxidant Activity Using 3T3 Fibroblast Cells

As Xyl-GA exhibited superior activity compared with Xyl and GA in various in vitro tests, its antioxidant activity was assessed in a cellular environment to determine whether Xyl-GA also functioned as an antioxidant in this setting. For this purpose, 3T3 cells (murine fibroblasts) were used as a model.

Given that the highest antioxidant activity of Xyl-GA was noted in the hydroxyl radical scavenging test, cellular exposure involved hydrogen peroxide. Within the cellular environment, hydrogen peroxide undergoes rapid decomposition, leading to the liberation of hydroxyl radicals. Since GA exhibited minimal activity in the hydroxyl radical scavenging test, its efficacy was not assessed in cellular experiments, nor in subsequent assays involving zebrafish, which are detailed following the cellular investigations.

**Figure 5:**
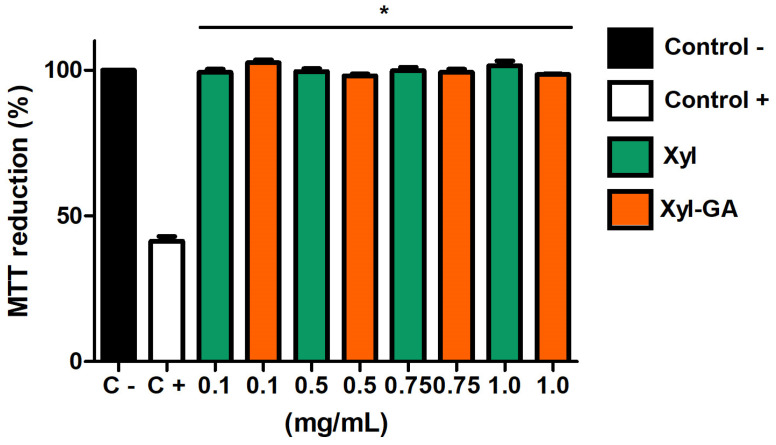
Effect of different concentrations of Xyl and Xyl-GA on the ability of 3T3 cells to reduce MTT. Control− corresponds to cells that were not exposed to H_2_O_2_. Control+ corresponds to cells exposed to H_2_O_2_ (500 µM, final concentration). * Represents significant differences between samples and Control+ (*p* < 0.001). There are no significant differences between the samples and Control−.

Initially, the samples were tested for their cytotoxicity towards these cells. As depicted in Figure 5, both Xyl and Xyl-GA did not impact the cells’ ability to reduce MTT. This suggests that these samples do not exhibit cytotoxicity at the evaluated concentrations.

The data observed in Figure 5 corroborates the findings demonstrated by Melo-Silveira [17]. These authors also showed that Xyl (at concentrations ranging from 0.01 to 2.0 mg/mL) does not exhibit cytotoxicity towards 3T3 cells. Furthermore, their research demonstrated that the addition of gallic acid to the xylan molecule did not render it cytotoxic under the tested conditions.

**Figure 6:**
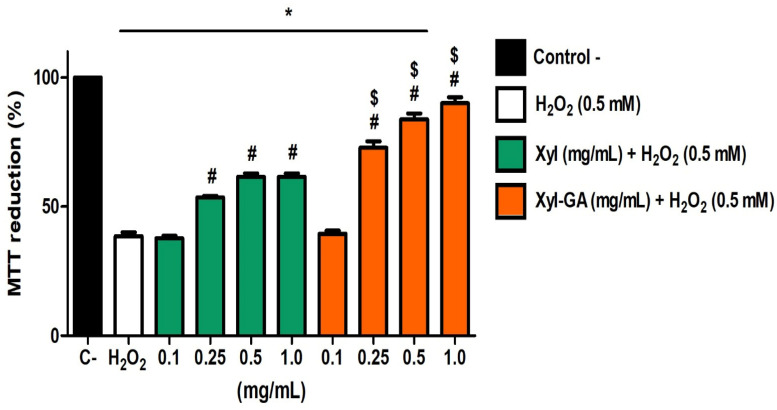
Effect of different concentrations of Xyl and Xyl-GA on the ability of 3T3 cells to reduce MTT in the presence of H_2_O_2_. Control− corresponds to cells that were not exposed to H_2_O_2_ (500 µM, final concentration). * Represents significant differences between the samples and H_2_O_2_ (*p* < 0.001). # Represents significant differences between the samples and Control− (*p* < 0.001). $ Represents significant differences between Xyl and Xyl-GA at the same concentration (*p* < 0.001).

As xylans did not exhibit cytotoxic activity toward 3T3 cells, the next step was to evaluate whether they could protect the cells from a stress-inducing condition. To investigate this, 3T3 cells were exposed to H_2_O_2_ (500 µM) in the presence and absence of various concentrations of xylans. The obtained data are presented in Figure 6. Cells that were not exposed to peroxide (negative control) could reduce MTT by approximately 100%. Conversely, the ability of cells exposed to peroxide to reduce MTT significantly decreased (*p* < 0.001) compared with the positive control, reaching only around 40% MTT reduction.

Both Xyl and Xyl-GA, at the lowest evaluated concentration (0.1 mg/mL), were unable to protect cells from the damage caused by H_2_O_2_. On the other hand, the protective effect of xylans was observed at other concentrations. However, Xyl-GA demonstrated superior protective capabilities compared with Xyl. When comparing the effects of these two xylans at the same concentration, the effect of Xyl-GA was significantly greater than that of Xyl (*p* < 0.001). It is noteworthy that Xyl-GA at a concentration of 1.0 mg/mL completely mitigated the cytotoxic effects of H_2_O_2_, as cells exposed to this concentration exhibited MTT reduction levels similar to those observed in the negative control group.

Data from the literature indicate that gallic acid can either protect [47,48] or not protect [49] cells from exposure to hydrogen peroxide, demonstrating that its action is cell-dependent. In the case of polysaccharides conjugated with gallic acid, this cell-dependent effect was not observed. However, it is worth noting that the referenced studies only evaluated the antioxidant action of these gallic acid-conjugated polysaccharides against a single cell line. Nevertheless, it can be concluded that some polysaccharides, when conjugated with gallic acid, lack the ability to shield cells from oxidative stress, as exemplified by laminarin conjugated with gallic acid [35], while others exhibit this protective capability, as observed with fucoidan derived from the alga *Spatoglossum schröederi* [28].

Xyl-GA demonstrated antioxidant activity in several in vitro tests, including the reducing power and total antioxidant capacity (TAC) tests, which were conducted at different pH levels. The cellular environment also exhibited substantial pH adaptability, and the data presented in Figure 6 suggest that the same attributes that rendered Xyl-GA a potent antioxidant in vitro persisted in the cellular environment. In the future, efforts will be directed toward identifying these characteristics to elucidate the mechanism of action of Xyl-GA. The data displayed in Figure 6 served as a basis for the subsequent in vivo studies mentioned below.

The final experiment conducted with cells aimed to determine the levels of intracellular reactive oxygen species (ROS). The objective was to assess whether ROS levels increased in the presence of peroxide and whether Xyl and Xyl-GA could reduce these levels. In this experiment, the xylans were exclusively evaluated at their highest concentration (1.0 mg/mL), as this concentration proved to be the most effective, as demonstrated in Figure 6.

**Figure 7:**
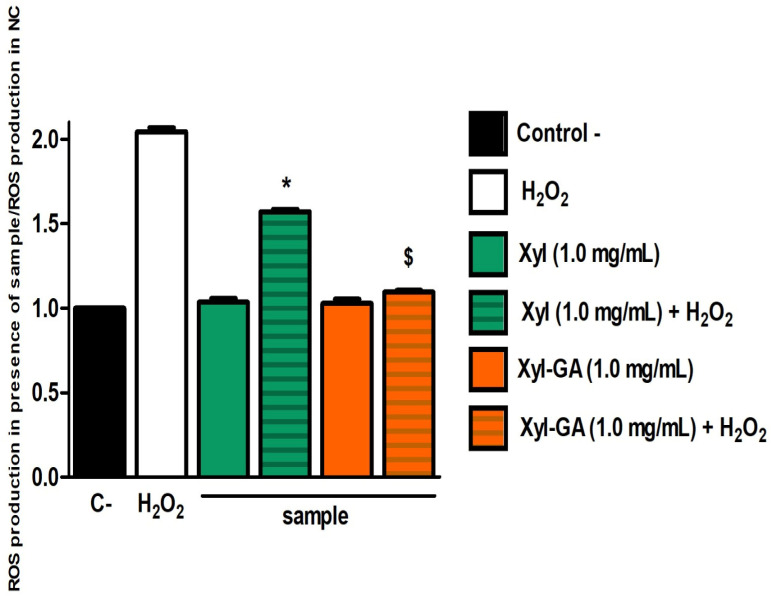
Production of reactive oxygen species (ROS) by 3T3 cells exposed to H_2_O_2_ (500 µM, final concentration). Control− corresponds to cells that were not exposed to H_2_O_2_. * Represents significant differences between the samples and Control− (*p* < 0.001). $ Represents significant differences between Xyl and Xyl-GA (*p* < 0.001). There is no significant difference between the comparison of C− vs. Xyl (1 mg/mL) and C− vs. Xyl-GA (1 mg/mL). The *p*-values for H_2_0_2_ vs. Xyl (1 mg/mL), vs. Xyl-GA (1 mg/mL), and vs. C− are all identical, with *p* < 0.001 in all cases.

In Figure 7, it is evident that ROS levels doubled when cells were exposed to hydrogen peroxide (500 µM). Conversely, when cells were exposed to xylans in the absence of peroxide, it was observed that the ROS levels were not significantly different from those observed in the negative control group. This suggests that, at least under the conditions tested, xylans do not induce oxidative stress in 3T3 cells.

When cells were exposed simultaneously to peroxide and xylans, it was observed that both Xyl and Xyl-GA significantly reduced the ROS levels within the cells. However, the effect of Xyl-GA was markedly superior to that of Xyl, as the ROS levels in cells treated with Xyl-GA were remarkably similar to the ROS levels in cells from the negative control group.

### 2.5. In Vivo Antioxidant Capacity

**Figure 8:**
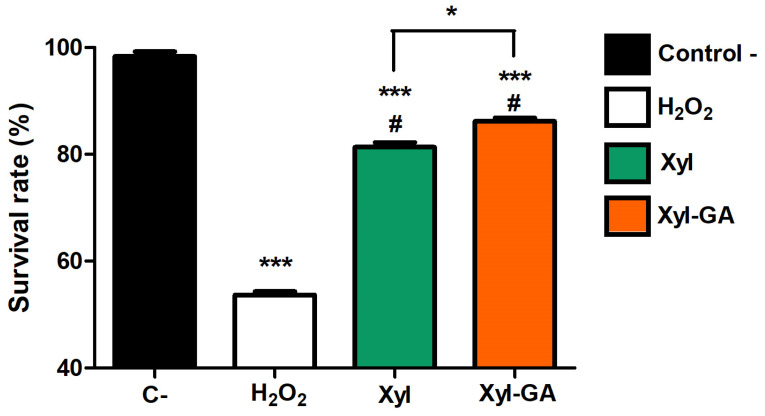
Protective effect of Xyl and Xyl-GA against oxidative stress in zebrafish (*Danio rerio*). *** *p* < 0.001 vs. C−; # *p* < 0.001 vs. positive control; * indicates a significant difference between the Xyl and Xyl-GA samples (*p* < 0.05).

In vitro and in-cell experiments demonstrated that combining Xyl with gallic acid resulted in the formation of Xyl-GA, which exhibited superior antioxidant activity. Subsequently, we evaluated the antioxidant potential of these compounds with in vivo analyses using a zebrafish model (*Danio rerio*), a vertebrate that shares biochemical and physiological characteristics with mammals, which has been used in recent research to identify new drugs and to evaluate the toxicological effects of pharmaceutical products [50]. The embryo survival rate after exposure to various compounds was evaluated, as illustrated in Figure 8.

Hydrogen peroxide proved to be highly toxic to the embryos, with only approximately 40% of them surviving after exposure to peroxide (Figure 8). In contrast, Xyl provided protection to the embryos against the effects of peroxide. However, it was with Xyl-GA that the highest rates of embryo survival were achieved. In this case, the number of surviving embryos was significantly greater than what was observed in the group treated with Xyl.

**Figure 9:**
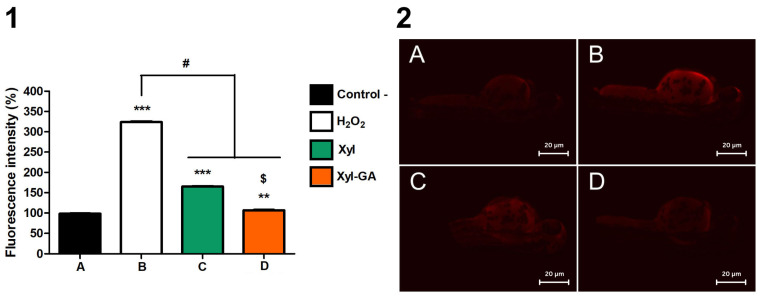
(**1**) Bar chart showing the ratio of embryo survival in different exposures, with its respective image (**2**) showing cell damage on zebrafish, where in redder areas, there are more dead cells on the live fish. The same letter corresponds to the same image and bar. (**A**): control. (**B**): peroxide. (**C**): peroxide + Xyl. (**D**): peroxide + Xyl-GA. *** *p* < 0.001 vs. C−; ** *p* < 0.01 vs. negative control; # *p* < 0.001 vs. positive control; $ indicates a significant difference between the Xyl and Xyl-GA samples (*p* < 0.001). The bars correspond to 20 µm.

After hatching, the larvae were stained with acridine orange, a fluorescent probe that labels nuclear compounds, revealing cells undergoing apoptosis. Figure 9(1) displays the fluorescence intensity of the larvae, which increased by approximately 3-fold after peroxide exposure. This intensity was significantly reduced in the presence of Xyl-GA, approaching the levels observed in the negative control (where no substance was added). In Figure 9(2), images of zebrafish larvae after fluorescence staining are presented.

It is noteworthy that the survival rate of the embryos exposed to peroxide and gallic acid (0.012 mg/mL) was significantly similar to that observed in the embryos treated with peroxide alone. In other words, gallic acid did not provide protection to the embryos against the effects of peroxide.

This likely occurred because gallic acid, similar to other phenolic compounds, has low bioavailability due to its limited absorption, rapid metabolism, and subsequent excretion [23,24]. Therefore, the data suggest that the conjugation of gallic acid with xylan increased its bioavailability in vivo. In the future, we plan to conduct experiments with a mammalian model to assess, among other factors, the concentration of gallic acid in animal blood. This measurement will help determine whether the conjugation with xylan indeed leads to an enhancement in the bioavailability of gallic acid.

In summary, the findings indicate that the antioxidant activity of corn cob xylan can be enhanced through conjugation with gallic acid. This suggests promising ways for the utilization of Xyl-GA in various sectors including cosmetics, food, and medicine. Such developments could lead to increased utilization of corn cobs, thereby mitigating waste.

## 3. Materials and Methods

### 3.1. Materials

Potassium ferricianyde, gallic acid, Folin–Ciocalteau reagent, ferrous sulfate II, and sulfuric acid were purchased from Merck (Darmstadt, Germany). Coomassie brilliant blue R-250, 2,2′,2″,2‴-(Ethane-1,2-diyldinitrilo) tetraacetic acid (EDTA), ascorbic acid, methionine, ammonium molybdate, and Dulbecco’s Modified Eagle Medium (DMEM), were purchased from Sigma-Aldrich Co. (St. Louis, MA, USA). Methanol, ethanol, acetone, acetic acid, and sulfuric acid were purchased from CRQ (São Paulo, SP, Brazil). Sterile fetal bovine serum—FBS was purchased from Cultilab (Campinas, SP, Brazil). Penicillin and streptomycin were obtained from Thermo Fisher Scientific (Waltham, MA, USA). All other solvents and chemicals were of analytical grade.

### 3.2. Extraction of Xylan from Corn Cobs

The xylan extraction procedure followed the protocol outlined by Viana et al. [25]. In brief, corn cob powder underwent ethanol treatment to remove lipids and pigments. Subsequently, the dried powder was subjected to xylan extraction under the following conditions: for every 1 g of powder, it was mixed with 25 mL of 1.8 M NaOH solution, and the mixture was sonicated at 200 W for 30 min with 5 min intervals. The entire process was conducted at 60 °C. Following centrifugation at 10,000× *g* for 15 min at 4 °C, the soluble fraction containing xylan was separated from the powder. The xylan was then dialyzed, lyophilized, and stored protected from light at room temperature for subsequent analyses.

### 3.3. Conjugation of GA (Gallic Acid) and Xyl (Xylan)

The conjugation of xylan (Xyl) and gallic acid (GA) was performed using the methods described by Paiva et al. [26]. Initially, 500 mg of Xyl was dissolved in a water and acetic acid solution (2% *v*/*v*). Subsequently, 1 mL of 1.0 M H_2_O_2_ and 0.054 g of ascorbic acid, were added to this solution. After 30 min, 1.4 mmol of GA was introduced into the reaction and incubated for 24 h. The solution was then subjected to centrifugation using an Amicon^®^ Ultra-15 centrifugal filter (Millipore, Burlington, MA, USA) with a 3 kDa cut-off until all unreacted GA was removed. The resulting GA-conjugated Xyl solution was designated as “Xyl-GA” and was freeze-dried for future use.

### 3.4. Dosage of Total Phenolic Compounds, Total Sugar, and Protein

The quantification of phenolic compounds after the covalent binding of GA to Xyl was performed using the Folin–Ciocalteau colorimetric method at 765 nm, and gallic acid was used as standard [27]. The total sugar and protein contents were determined as described earlier [25]. The quantity of proteins, sugars, and phenolic compounds were combined, constituting 100%, and the percentage content of each element was calculated relative to this total sum.

### 3.5. Assessment of Reducing Power

To evaluate the reducing power of the samples, they were incubated for 20 min at 50 °C in phosphate buffer (0.2 M, pH 6.6) containing potassium ferricyanide (1%). The reaction was terminated using 10% trichloroacetic acid (TCA), followed by the addition of ferric chloride (0.1%). Absorbances were measured at 700 nm, and the results were expressed as a percentage. Ascorbic acid (0.2 mg/mL) served as the reference for 100% activity [27].

### 3.6. Evaluation of Hydroxyl (OH) Radical Scavenging

The hydroxyl radicals were generated using 3 mL of sodium phosphate buffer (150 mM, pH 7.4) containing 10 mM FeSO_4_·7H_2_O, 10 mM EDTA, 2 mM sodium salicylate, and 30% H_2_O_2_, along with varying concentrations of Xyl and Xyl-GA. The radicals were produced via the Fenton reaction (Fe^2+^ + H_2_O_2_ → Fe^3+^ + OH− + OH), and the negative control consisted of phosphate buffer without hydrogen peroxide. The presence of hydroxyl radicals was quantified at 510 nm following incubation at 37 °C for 1 h [27]. 

### 3.7. Copper-Chelating Ability

The capacity of the samples to chelate copper ions was assessed following the method outlined by Domazetovic et al. [51]. Pyrocatechol violet, utilized as the reagent in this assay, has an affinity for various cations including copper. In the presence of chelating agents, the formation of this complex is impeded, resulting in diminished coloration. This decrease in color intensity facilitates the determination of copper ion chelating activity exhibited by samples. The assay was conducted in 96-well microplates, where the reaction mixture consisted of different concentrations of the samples, pyrocatechol violet (4 mM), and copper II sulfate pentahydrate (50 mg/mL). Each well’s solution was thoroughly mixed using a micropipette, and the absorbance was subsequently measured at 632 nm. The chelating effect was determined using the following formula, where the blank denotes the absorbance in the absence of chelating agents

Chelating Effect (%) = (^A^control − ^A^sample/^A^control) × 100 (1)

### 3.8. Determination of Total Antioxidant Capacity

This assay relies on the ability of samples to reduce Mo(VI) to Mo(V), followed by the formation of a green phosphate/Mo(V) complex under acidic conditions [52]. Tubes containing samples and a reagent solution composed of 0.6 M sulfuric acid, 28 mM sodium phosphate, and 4 mM ammonium molybdate were incubated at 95 °C for 90 min. Subsequently, after cooling the mixture to room temperature, the absorbance of each solution was measured at 695 nm against a blank. The concentration at which the absorbance values ceased to increase was determined post-experimentation. In the case of Xyl, GA, and Xyl-GA polysaccharides, no further changes in absorbance values were observed beyond a concentration of 0.1 mg/mL. Therefore, this concentration was selected to calculate the total antioxidant capacity (TAC), as previously described [27]. Ascorbic acid served as the standard in this assay.

### 3.9. MTT (3-(4,5-Dimethylthiazol-2-yl)-2,5-diphenyltetrazolium Bromide) Assay

For the experiments, a culture of 4 × 10^3^ cells (3T3 murine fibroblast cells, ATCC n° NIH/3T3—CRL-1658) was established in 96-well plates using DMEM (Sigma-Aldrich Co., St. Louis, MA, USA). The culture medium contained varying concentrations of xylans (ranging from 0.01 to 0.1 mg/mL) or GA (ranging from 0.01 to 0.1 mg/mL). The cells were incubated for 24 h at 37 °C with 5.0% CO_2_, with six replicates for each concentration. In the case of GA, the plates were maintained under these conditions while being shielded from light. The cell’s ability to reduce MTT (Sigma-Aldrich Co., St. Louis, MA, USA) using the colorimetric MTT assay was determined by following a previously described procedure [27].

### 3.10. Induced Oxidative Stress Assay

The 3T3 cells (1 × 10^6^ cells/mL) were cultured in 6-well plates using Dulbecco’s Modified Eagle Medium (DMEM) supplemented with 10% fetal bovine serum (FBS); with each well containing 1 mL of the medium. After a 24-h incubation period, the plates were washed, and a fresh mixture of 1 mL DMEM (with 10% FBS), xylans (1.0 mg/mL), and H_2_O_2_ (500 µM, final concentration) was added. The cells were then maintained under standard culture conditions (37 °C, 5% CO_2_, in darkness) for 6 h. Subsequently, the medium was replaced with 1 mL of fresh medium. After an additional 24 h, the cells were subjected to the MTT assay.

### 3.11. Intracellular ROS Production

To assess intracellular levels of reactive oxygen species (ROS), we utilized 2′,7′-dichlorofluorescein (DCFH), the oxidized form of 2′,7′-dichlorofluorescein diacetate (DCFH-DA) (procured from Sigma Chemical Company, St. Louis, MO, USA). In 24-well plates, 3T3 cells (3 × 10^5^ cells/well) were cultured for 24 h in DMEM supplemented with fetal calf serum (FCS), washed, and subsequently incubated in DMEM supplemented with 10% FBS, along with H_2_O_2_ (500 µM, final concentration), Xyl, and Xyl-GA, each at a concentration of 1.0 mg/mL.

As a control, a negative control group was established using DMEM supplemented with 10% FBS alone. The plates were maintained under standard culture conditions (37 °C, 5% CO_2_, in darkness) for 6 h. Following the treatment, the supernatant was removed, the cells were washed with phosphate-buffered saline (PBS), and 100 µM DCFH-DA in DMEM containing 1% FBS was added. This mixture was then incubated at 37 °C for 2 h. Subsequently, DCFH-DA was removed, the cells were washed twice with PBS, and the fluorescence emitted was quantified using a flow cytometer (FACS Canto II, BD Biosciences, Eugene, OR, USA) equipped with FACSDiva software, version 6.1.2 (Becton Dickson, Franklin Lakes, NJ, USA).

Data analysis was performed using FlowJo software version vX.0.7 1997–2014 (FlowJo, Ashland, OR, USA), and the results were expressed as the ratio of ROS levels observed in cells exposed to the samples compared to ROS levels in cells from the negative control group (cells exposed only to medium).

### 3.12. Zebrafish Embryo Development

The wild strain of zebrafish (*Danio rerio*) was used as the animal model. The fish were bred in a controlled domestic environment. Reproduction was conducted at a ratio of two males to one female, with the fish placed in four separate breeding tanks. Interactions among the animals were facilitated both chemically and visually, with the fish separated by a partition during the night. Mating occurred for a duration of 1 h in the morning after removing the divider, ensuring precise fertilization timing and allowing for adequate testing periods. Following fertilization, the eggs were collected and placed in a plastic tray with water for 8 h. The photoperiod for the experiment was set at 24 h (12 h of darkness and 12 h of light). All experimental procedures were approved by the Committee for the Use of Animals at the Federal University of Rio Grande do Norte (CEUA 004002/2017).

### 3.13. Embryo Death Analysis after H_2_O_2_-Induced Oxidative Stress

We used the methodology recommended by Kim et al. [53] with modifications. The embryos were transferred to a 24-well plate containing 2.0 mL of pond water. Subsequently, they were incubated with GA (0.074 mg/mL) and Xyl and Xyl-GA (both 0.1 mg/mL) for 1 h, and then H_2_O_2_ (500 µM, final concentration) was added to this solution. The embryos were incubated for 24 h at 28 °C, with the H_2_O_2_ solution replaced by stock water. The time and temperature conditions were then repeated. The process was carried out without the interference of light. The protective effects of the compounds were evaluated by counting the number of surviving embryos.

### 3.14. Statistical Analyses

All data are expressed as the mean ± standard deviation (*n* = 3) from three observations. Statistical analysis was performed by one-way ANOVA followed by Student’s *t*-test. Statistical significance was set at *p* < 0.05. Analyses were performed using GraphPad Prism software version 9.

## 4. Conclusions

This marks the first report on the production of xylans conjugated with gallic acid. In this study, it was discovered that the conjugation of xylan with gallic acid significantly enhanced the antioxidant activity of this compound, both in in vitro, in-cell assays, and particularly, in vivo using the zebrafish model. Xyl-GA exhibited higher antioxidant activity compared with Xyl, as demonstrated by the results of four in vitro antioxidant assays. In addition, Xyl-GA displayed cytotoxicity toward 3T3 cells. Among them, Xyl-GA provided the most effective protection to 3T3 cells against H_2_O_2_-induced stress, achieved through the suppression of ROS production. In addition, Xyl-GA was able to inhibit the damage caused by H_2_O_2_ in zebrafish. Overall, our data suggest that Xyl-GA holds potential therapeutic value for addressing disturbances related to oxidative stress. These findings suggest that other pharmacological activities of xylan may also be enhanced through the gallic acid modification process. Moreover, further investigations are necessary to determine the molecular structure of the new compound and establish correlations with its pharmacological activities.

## Figures and Tables

**Table 1:** Comparison of dosage of sugar, proteins, and phenolic compounds between Xyl and Xyl-GA.

Sample	Sugar (%)	Protein (%)	Phenolic Compounds (%)
Xyl	97.4 ± 1.5	<1	0.8 ± 0.1
Xyl-GA	95.5 ± 0.3	<1	2.4 ± 0.1 *

* Indicates a significant difference (*p* < 0.05).

## Data Availability

Data are contained within the article.
